# Quantum chemical calculations of tryptophan → heme electron and excitation energy transfer rates in myoglobin

**DOI:** 10.1002/jcc.24793

**Published:** 2017-04-01

**Authors:** Christian J. Suess, Jonathan D. Hirst, Nicholas A. Besley

**Affiliations:** ^1^ School of ChemistryUniversity of Nottingham, University ParkNottinghamNG7 2RDUnited Kingdom

**Keywords:** myoglobin, electron transfer, excitation energy transfer, TDDFT

## Abstract

The development of optical multidimensional spectroscopic techniques has opened up new possibilities for the study of biological processes. Recently, ultrafast two‐dimensional ultraviolet spectroscopy experiments have determined the rates of tryptophan → heme electron transfer and excitation energy transfer for the two tryptophan residues in myoglobin (Consani et al., Science, 2013, 339, 1586). Here, we show that accurate prediction of these rates can be achieved using Marcus theory in conjunction with time‐dependent density functional theory. Key intermediate residues between the donor and acceptor are identified, and in particular the residues Val68 and Ile75 play a critical role in calculations of the electron coupling matrix elements. Our calculations demonstrate how small changes in structure can have a large effect on the rates, and show that the different rates of electron transfer are dictated by the distance between the heme and tryptophan residues, while for excitation energy transfer the orientation of the tryptophan residues relative to the heme is important. © 2017 The Authors. Journal of Computational Chemistry Published by Wiley Periodicals, Inc.

## Introduction

Electron transfer (ET) and the transfer of excitation energy are fundamental processes in biological systems. The efficient and controlled movement of electrons is one of the primary regulation mechanisms in biology and critical for the existence of living organisms,^1^, ^2^ and excitation energy transfer (EET) is important in light harvesting systems.^3^ This has motivated the development of experimental and computational approaches to characterize the mechanisms of ET and EET processes. However, these studies are challenging due to the fast time‐scale of ET and EET and the complexity of biological systems. Two‐dimensional ultraviolet (2D‐UV) spectroscopy has emerged as a powerful technique for the study of biological systems, as many biological chromophores, such as tryptophan, absorb in this region of the spectrum.^4^ Alongside these experimental techniques complementary theoretical approaches have been developed,^5^ which can play an important role in interpreting spectra measured in experiment. Here, we focus on tryptophan →heme ET and EET in myoglobin. Myoglobin, an oxygen carrier in muscle tissue, comprises a single polypeptide chain of 153 amino acids arranged in eight *α*‐helices with an iron porphyrin active site and has been described as the hydrogen atom of biology and a paradigm of complexity.^6^

Myoglobin has two tryptophan residues (Trp7 and Trp14), and the fluorescence decay times of these residues have been measured in several myoglobin complexes and are approximately 120 ps for Trp7 and 20 ps for Trp14.^7^, ^8^, ^9^, ^10^ Recently, Chergui and coworkers studied myoglobin with ultrafast 2D‐UV spectroscopy providing insight into the ET and EET processes from the tryptophan residues to the heme.^8^ The tryptophan residues are located in an *α* helix separated from the heme by the E helix, of which several amino acids (Val68, Leu69, Thr70, Gly74, Ile75, and Leu76) lie within the direct path to the heme. The experiments showed that Trp14, which is closer to the heme, decays predominantly by ET, whereas Trp7 relaxes by EET. The rate of ET was quantified, with the relaxation time for Trp14 determined to be ≤40 ps, with a much slower time of >40 ns for Trp7. Furthermore, a time of 140 ps was measured for EET from Trp7. The distance between the tryptophan and heme suggests the EET occurs via the Förster mechanism. Subsequent work on deoxy‐myoglobin indicated that a similar ET process is present in ligated ferrous myoglobins.^9^ It has been suggested that the ET pathway involves the Leu69 residue which is in van der Waals contact with the Trp14 and Val68 residues,^9^ although other work has found a glutamic acid residue to be important.^11^

It is important that computational modeling of ET and EET develops alongside advances in experiments to predict rates of ET and EET with sufficient accuracy to elaborate on the detailed underlying mechanisms. In particular, computational modeling of these processes can provide an understanding of the different ET and EET properties of the two tryptophan residues. The theoretical treatment of ET usually follows Marcus theory^12^, ^13^, ^14^ wherein for weak coupling the rate is expressed as

(1) $$k_{\text{ET}}=\frac{|V_{\text{DA}}{|}^{2}}{\hbar }\sqrt{\frac{\pi }{\lambda k_{B}T}}\exp\left[-\frac{{\left(\lambda +\Delta G\right)}^{2}}{4\lambda k_{B}T}\right]\;$$

where ΔG is the change in free energy between the final and initial states, *λ* is the reorganization energy and corresponds to the energy for ET in the absence of a change in structure and *V*_DA_ is the diabatic electron coupling matrix element between the two electronic states. Within this formalism the rate can be estimated once the key quantities, ΔG, *λ*, and *V*_DA_ have been determined, and this approach has been successfully applied to many systems.^15^, ^16^, ^17^ Many heme proteins, such as cytochrome c, are involved in biologically important ET reactions. In contrast to cytochrome c, the heme group in myoglobin is not covalently bound and metal substitution is easier to effect, which has motivated many experiments on myoglobin as an exemplar of ET in heme proteins.^18^ The quantitative determination of the rates of ET and EET in myoglobin provides an opportunity to assess computational models for determination of these rates. This can establish the important criteria for calculation of these rates which can then be applied in studies of ET and EET of biologically important processes.

A range of computational methods of varying degrees of sophistication have been proposed for the study of ET, and comprehensive review articles on this subject are available.^19^, ^20^, ^21^ One relatively simple but successful approach is the pathway tunneling model of Beratan and coworkers.^22^, ^23^ This model provides a framework to characterize the influence of the protein structure on *V*_DA_ in biological ET reactions. We use this approach here as a starting point for more sophisticated time‐dependent density functional theory (TDDFT) calculations and as a benchmark. The model assumes a donor‐acceptor complex mechanism and the ET is mediated by consecutive interactions between atoms connecting the donor and acceptor. The steps taken are characterized by decay factors or “penalties” associated with covalent bonds, hydrogen bonds, or through‐space jumps. Covalent‐bond mediated steps are deemed to have a low tunneling barriers and hence assigned a low penalty, whilst higher penalties are applied to those with high tunneling barriers such as a through‐space jump. Using a graph‐search algorithm^24^ to determine all potential pathways, the overall penalty for ET is the product of each penalty throughout every step^25^

(2) $$V_{\text{DA}}=A\prod_{i}\varepsilon_{i}^{C}\prod_{j}\varepsilon_{j}^{H}\prod_{k}\varepsilon_{k}^{S}$$

where $\varepsilon_{i}^{C}$ is the penalty for propagation through covalent bond *i*, $\varepsilon_{j}^{H}$ is the penalty for propagation through a hydrogen bond *j* and $\varepsilon_{k}^{S}$ is the penalty for the through‐space jump *k*. *A* is the pre‐factor, and in this work we take *A* = 1 eV.^26^ Using this approach, it is possible to identify the strongest ET pathways between the donor and the acceptor, estimate partial electronic couplings mediated by each pathway, calculate the importance of individual protein groups for mediating ET, and ultimately determine the most dominate ET pathway between the donor and acceptor.

The direct calculation of the electron coupling with quantum chemical methods provides a potentially more accurate means for determining the coupling strength. There is a wide range of approaches to this problem including different schemes to characterize the diabatic states^19^, ^27^, ^28^, ^29^ along with the choice of electronic structure method. The coupling elements for ET can be evaluated using the Generalized Mulliken‐Hush (GMH) scheme^30^

(3) $$V_{\text{DA}}^{\text{ET}}=\frac{\left(E_{2}-E_{1})|{\vec{\mu }}_{12}\right|}{\sqrt{{\left({\vec{\mu }}_{11}-{\vec{\mu }}_{22}\right)}^{2}+4|{\vec{\mu }}_{12}{|}^{2}}}$$

where ${\vec{\mu }}_{11},\;{\vec{\mu }}_{22}$, and ${\vec{\mu }}_{12}$ represent the permanent and transition dipole moments for the two states.

An advantage of a quantum chemistry‐based approach is that it is also possible to study the rates of EET, and an overview of the theoretical treatment of EET is available.^31^ The coupling elements associated with EET can be calculated through the fragment excitation difference (FED) approach.^27^

(4) $$V_{\text{DA}}^{\text{EET}}=\frac{(E_{2}-E_{1}){\bar{\Delta x}}_{12}}{\sqrt{{\left(\Delta x_{11}-\Delta x_{22}\right)}^{2}+4{\bar{\Delta x}}_{12}^{2}}}$$

where Δx represents elements of the excitation difference matrix. Here, we are concerned with EET occurring over long range through dipole‐dipole coupling in a Förster mechanism. Computational methods for the calculation of EET via a Dexter mechanism, which requires overlap of the wavefunctions of the donor and acceptor, have been described elsewhere.^32^

Evaluating the terms in eq. (1) requires excited state electronic structure methods, including structural optimization of the relevant states. This is commonly done using methods such as single excitation configuration interaction (CIS) or TDDFT.^19^, ^33^, ^34^, ^35^, ^36^, ^37^, ^38^, ^39^ The study of large biological systems is challenging, as the size of the system usually precludes a full treatment with quantum chemical methods, necessitating some further approximations. Despite this inherent complexity, several studies have reported calculations of ET in proteins based on Marcus theory.^40^ One approach has been to compute coupling matrix elements for structures extracted from a molecular dynamics simulation.^41^, ^42^, ^43^ Recent work has studied the ultrafast ET in cryptochromes based on GMH coupling strength computed using TDDFT.^44^ Furthermore, the absorption and fluorescence spectra of tryptophan residues embedded in the protein environment have been studied using TDDFT calculations.^45^, ^46^ Studies of EET of large biological systems have also been reported.^47^, ^48^, ^49^ This includes application of the FED approach with TDDFT to study the electronic energy transfer pathways in cyanobacteria phycocyanin^48^ and cyanobacteria allophycocyanin.^49^

In this study, we aim to provide insight into the structural factors that affect the rates of both ET and EET in the protein myoglobin. The availability of experimental data for the rates of tryptophan → heme ET and EET allows the accuracy of TDDFT‐based calculations to be assessed. Treating ET and EET in an equivalent way, and achieving good agreement with experiment observations, provides a sound basis for the physical basis for the ET and EET rates observed in experiment to be explored with confidence.

## Methodology

The donor‐acceptor coupling elements, *V*_DA_, for the both Trp14 →heme and Trp7 →heme ET processes were computed using the pathways tunneling method based on the coordinates of the crystal structure (PDB: 1YMB) using the software of Balabin et al.^24^ The electron coupling was also evaluated using TDDFT with the GMH scheme for ET and the FED approach for EET. The TDDFT calculations, using the long‐range corrected CAM‐B3LYP exchange‐correlation functional,^50^ were performed on reduced structural models based on the crystal structure coordinates. The location of hydrogen atoms were constructed using the IQMOL software.^51^ Key intervening residues that were identified based on the pathways tunneling calculations were included in the TDDFT calculations. To explore the sensitivity of the computed coupling to fluctuations in structure, further couplings were computed for a range of crystal structures (PDB: 1BZR, 2MB4, 4MBN, 1YMB) including those recorded in time‐resolved serial femtosecond crystallography experiments (PDB: 5CN4 at 0, 50, and 150 ps).^52^

**Figure 1:**
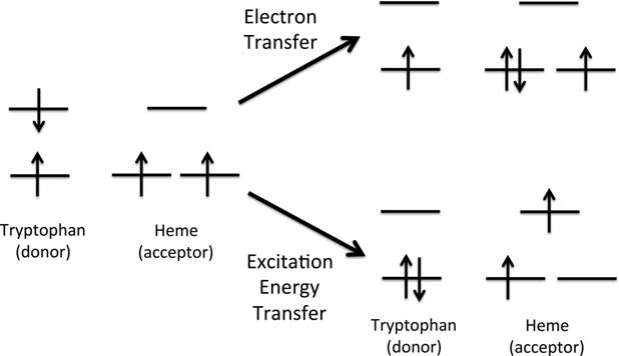
Schematic of the electronic configurations for ET and EET processes from the *S*_1_ state of a tryptophan residue to the heme.

In the 2D‐UV experiment, ET or EET transfer occurs following the initial electronic excitation of the tryptophan. Here, we study the ET from the tryptophan residues to the porphyrin ring to form an Fe(II)‐porphyrin *π*‐anion radical. Subsequent ET from the ring to iron to form the ferric heme is expected to be sufficiently fast that it does not affect the overall rate. A schematic of the electronic states involved is given in Figure 1. It shows that to describe the ET and EET processes requires calculations on the ground state, local excited state of the tryptophan, local excited state of the heme and a tryptophan →heme charge transfer (CT) excited state. The accurate description of the electronic structure of metal‐porphyrin systems is a challenge for computational methods, where the prediction of the correct ground state electronic configuration is debated.^56^, ^57^, ^58^ Multiconfigurational perturbation theory‐based calculations in conjunction with large basis sets represent the most reliable methods, however, the computational cost of these methods makes them unsuitable for the current study and the calculations presented here are based on DFT and TDDFT. These calculations find the lowest energy spin state of the iron porphyrin ring to be a triplet state, which is computed in our work to lie about 1.8 eV lower in energy than the singlet state at the TDDFT level of theory. This is consistent with previous DFT studies.^58^ The excited states arise from local excitations between the highest occupied molecular orbital (HOMO) and lowest unoccupied molecular orbital (LUMO) of the tryptophan and a CT excitation from the HOMO of tryptophan to the heme. These orbitals correspond to *π* and $\pi^{*}$ orbitals localized on the tryptophan residues or the heme. Table 1 shows excitation energies for the relevant states computed with CIS and a range of exchange‐correlation functionals that are suitable for describing CT excitations for a model system that includes the two tryptophan residues and the heme in their geometries taken directly from the crystal structure. The CAM‐B3LYP functional predicts excitation energies for the HOMO → LUMO transitions of Trp14 and Trp7 to occur at 4.12 eV and 4.03 eV, respectively, which are consistent with the value of 4.3 eV measured for tryptophan in the gas phase.^53^ The HOMO → LUMO transition localized on the heme lies at 2.37 eV. This transition corresponds to the Q band in porphyrin related compounds, which lies in the region 1.9–2.2 eV.^54^, ^55^ CAM‐B3LYP performs best of the functionals considered, particularly for Q band excitation of the heme.

**Table 1:** Computed excitation energies in eV with CIS and TDDFT with different exchange‐correlation functionals.

HOMO → LUMO transition	CIS	M06‐HF	*ω*B97‐X	CAM‐B3LYP	Exp.^a^
^14^Trp →^14^Trp	4.39	4.29	4.73	4.12	4.30
^7^Trp →^7^Trp	4.20	3.99	4.52	4.03	4.30
Heme → Heme	3.60	2.84	2.96	2.37	1.9–2.2
^14^Trp → Heme	3.52	3.64	3.78	4.06	
^7^Trp → Heme	3.44	3.55	3.74	3.93	

a Experimental data from Refs. [53, 54, 55].

All calculations were performed with Q‐CHEM^59^ and used the 6‐31G* basis set. Calculations using larger basis set 6‐311G* did not show significant difference in the computed values for the smaller systems studied here, and hence, the less demanding basis set was used. This observation is consistent with previous simulations of the electronic spectra of porphyrin.^60^

**Figure 2:**
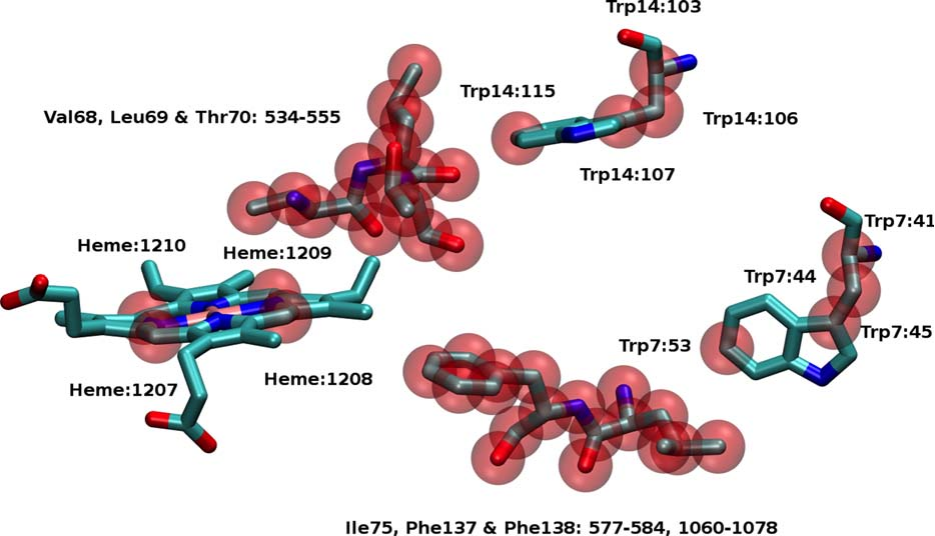
Atoms highlighted in red are held fixed in position during the optimization of the structure of the heme, intervening residue and tryptophan. [Color figure can be viewed at wileyonlinelibrary.com]

One of the most computationally challenging aspects of the application of Marcus theory is the structural optimization of the initial and final states. These states are electronically excited states, and in our calculations we assessed optimizing the structures using several methods: CIS, TDDFT, and the Maximum Overlap Method (MOM).^61^ The *S*_1_ excited state geometries for the heme predicted by the three excited state methods, CIS, TDDFT, and MOM, show a largest RMSD between any two structures of 0.04 Å. MOM provides accurate predictions for excited state structures^62^ and was chosen as the preferred method of excited state optimization, as it was the most straightforward to apply. When optimizing the structure of high‐lying excited states with TDDFT or CIS, in many instances the order of the roots changed between optimization cycles making it necessary to monitor the nature of the excited states constantly to ensure that the correct state was being optimized. An unconstrained optimization of the reduced model of the protein would yield an unphysical structure. Consequently, it is necessary to constrain the optimization in some way to maintain a biologically realistic geometry for the residues within the protein while allowing for some relaxation of the structure. These constraints represent the physical constraints that the surrounding protein environment would impose on the fragments. During the optimization a subset atoms, shown in Figure 2 and listed in Table 2, were kept frozen in position. Under the constraint of these frozen atoms, the two tryptophans are kept a fixed distance from the heme consistent with the crystal structure, but allowed sufficient freedom for relaxation in the optimization process. The constraints also restrict the macrocyclic of the heme from becoming unduly distorted while allowing porphyrins to dome and ruffle in their excited states.^63^, ^64^ The methodology described does not take into account the effects of entropy in ΔG and neglects the role of solvent. These effects may be significant, but to describe them accurately at a quantum chemical level is beyond our current capabilities.

**Table 2:** Atoms frozen in the geometry optimizations.

Residue	Index in PDB (1YMB)
Trp7	41,44,45,53
Trp14	103,106,107,115
Val68	534–540
Leu69	541–548
Thr70	549–555
Ile75	577–584
Phe137	1060–1067
Phe138	1068–1078
Heme	1207,1208,1209,1210

## Results and Discussion

**Figure 3:**
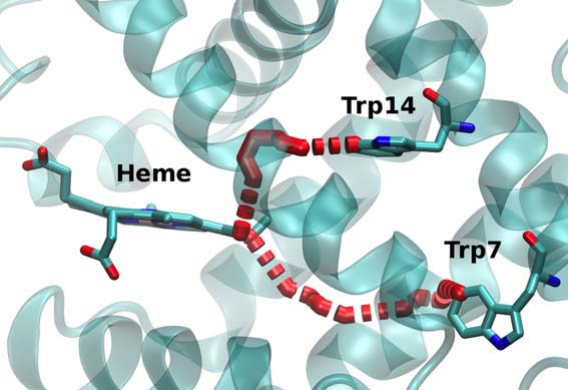
Dominant electron transfer pathways for Trp14 and Trp7 to heme calculated by the pathway tunneling model. For details of the residues see the main text. [Color figure can be viewed at wileyonlinelibrary.com]

Figure 3 shows the strongest ET pathways predicted by the pathways tunneling model for ET between the tryptophan residues and the heme. The computed pathways for the two tryptophans pass through different parts of the E helix, where key amino acids are Val68 for Trp14 and Ile75 for Trp7. The computed values of the electron coupling term $\left(\left|V_{\text{DA}}\right|\right)$ are $8.82\times 10^{-3}$ eV and $4.27\times 10^{-4}$ eV for Trp14 and Trp7, respectively. These values are consistent with the observation from experiment that ET from Trp14 occurs on a faster timescale than for Trp7.

**Figure 4:**
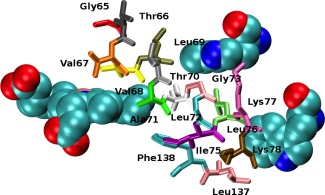
The reduced myoglobin system consisting of heme, Trp14, Trp7, and the E‐Helix. Different colors represent each amino acid. [Color figure can be viewed at wileyonlinelibrary.com]

Tables 3 and 4 show the computed coupling elements for structural models that incorporate different components of the ET pathway that were identified by the pathways tunneling model calculation. The TDDFT coupling strengths are computed using the GMH scheme, and correspond to the initial state being the *S*_1_ excited state of the tryptophan residue. The tunneling pathways model used here makes no distinction between the strength of coupling between the donor in different electronic states, and we will examine the significance of this later. For some of the reduced structural models, the dominant pathway is broken resulting in a vacuum tunneling pathway with no significant coupling. The simplest structural model includes only the donor tryptophan and heme. However, intervening residues are likely to affect the computed value for the coupling. We study the importance of the intervening residues by including a single residue (Val68 for Trp14 and Ile75 for Trp7), three residues (Val68, Leu69 and Thr70 for Trp14; Ile75, Leu137, and Phe138 for Trp7) and the full E helix with the additional Leu137 and Phe138 residues between the tryptophan and heme. These residues are shown in Figure 4. The calculations show that the inclusion of just a single key residue, Val68 for Trp14 and Leu137 for Trp7, (denoted Trp + Heme + 1AA in the table) has a significant affect on the computed coupling, with a three‐ to five‐fold increase in the strength of coupling. This indicates that Val68 and Ile75 are important in facilitating ET to the heme. The inclusion of further structural elements leads to much smaller additional increases in the coupling.

**Table 3:** Computed pathways model ( $\left|V_{\text{DA}}^{\text{Beratan}}\right|$) and TDDFT ( $\left|V_{\text{DA}}^{\text{TDDFT}}\right|$) coupling values in eV for Trp14 → heme electron transfer for different structural models.

Model	$\left|V_{\text{DA}}^{\text{TDDFT}}\right|$	$\left|V_{\text{DA}}^{\text{Beratan}}\right|$
Full Protein		$8.82\times 10^{-3}$
Trp14 + Heme + E helix	$7.92\times 10^{-3}$	$8.72\times 10^{-3}$
Trp14 + Heme + 3AA (Average)	$8.14\times 10^{-3}$	$7.71\times 10^{-3}$
Trp14 + Heme + 3AA	$6.92\times 10^{-3}$	$6.16\times 10^{-3}$
Trp14 + Heme + 1AA	$6.91\times 10^{-3}$	$3.65\times 10^{-3}$
Trp14 + Heme	$2.29\times 10^{-3}$	–

3AA and 1AA indicate three and one intervening amino acid residue included in the calculation, see text for details. The average values are evaluated using seven different crystal structures with the Trp14 + Heme + 3AA structural model.

**Table 4:** Computed pathways model ( $\left|V_{\text{DA}}^{\text{Beratan}}\right|$) and TDDFT ( $\left|V_{\text{DA}}^{\text{TDDFT}}\right|$) coupling values in eV for Trp7 → heme electron transfer for different structural models.

Model	$\left|V_{\text{DA}}^{\text{TDDFT}}\right|$	$\left|V_{\text{DA}}^{\text{Beratan}}\right|$
Full Protein		$4.27\times 10^{-4}$
Trp7 + Heme + E helix	$4.94\times 10^{-4}$	$3.69\times 10^{-4}$
Trp7 + Heme + 3AA (Average)	$6.62\times 10^{-4}$	$5.91\times 10^{-4}$
Trp7 + Heme + 3AA	$4.87\times 10^{-4}$	$3.29\times 10^{-4}$
Trp7 + Heme + 1AA	$4.82\times 10^{-4}$	–
Trp7 + Heme	$9.46\times 10^{-5}$	–

3AA and 1AA indicate three and one intervening amino acid residue included in the calculation, see text for details. The average values are evaluated using seven different crystal structures with the Trp7 + Heme + 3AA structural model.

The computed TDDFT $\left|V_{\text{DA}}\right|$ for the structural models with three intervening residues are $6.92\times 10^{-3}$ eV and $4.87\times 10^{-4}$ eV for Trp14 and Trp7, respectively. When the donor is the tryptophan in its ground (*S*_0_) state, the corresponding values computed with the same structural model are $6.75\times 10^{-3}$ eV and $4.10\times 10^{-4}$ eV. Thus, there is an increase in $\left|V_{\text{DA}}\right|$ in the excited state compared to the ground state. The calculation of $\left|V_{\text{DA}}\right|$ for the excited state adds significantly to the cost of the TDDFT calculation, as it requires higher energy roots in the TDDFT calculation to be computed. These results suggest that using the coupling value for the ground state is a reasonable approximation for the excited state, but is likely to underestimate the value for $\left|V_{\text{DA}}\right|$.

The calculated coupling strengths can be sensitive to changes in the structure. Average values for the coupling computed over seven different crystal structures reported in the literature with the Trp + Heme + 3AA structural model are also shown. The magnitude of the computed couplings vary by at most a factor of two, and the average values are reasonably close to the single structure values. For all structures, the qualitative difference between the computed couplings for the two tryptophan residues is observed, and average values of $8.14\times 10^{-3}$ eV and $6.62\times 10^{-4}$ eV are obtained for the TDDFT calculations. The ratio $\left|V_{\text{DA}}\right|$(Trp14): $\left|V_{\text{DA}}\right|$(Trp7) is found to be 12.3 from the TDDFT calculations, consistent with the value of 13.0 from the Beratan model.

It is common to describe the strength of electronic coupling for ET as an exponential dependence on the distance between the donor and acceptor^65^

(5) $$V_{\text{DA}}(r)=V_{\text{DA}}^{0}(r_{0})\exp\left[-\frac{\beta }{2}\left(r-r_{0}\right)\right]$$

where *r*_0_ is the van der Waals contact distance, and *β* is a parameter reflecting the effectiveness of the protein in mediating ET and typically ranges from 1.10 to 1.65 Å^−1^ for condensed phase systems and from 3 to 5 Å^−1^ for electron tunneling across a vacuum.^1^ Using the values of $\left|V_{\text{DA}}^{\text{TDDFT}}\right|$ for Trp14 and Trp7 computed here gives a value for *β* of 0.8 Å^−1^. This is close to typical values for this parameter for condensed phase systems, and suggests that the protein is effectively mediating ET and the slower rate of ET for Trp7 is associated largely with its greater distance from the heme (22.6 Å compared with 15.9 Å for Trp14 for the 1YMB crystal structure).

$\left|V_{\text{DA}}\right|$ for EET have been computed using the FED scheme with three intervening residues (Trp + Heme + 3AA) for both the *S*_0_ and *S*_1_ initial states of tryptophan. These values and the corresponding values for ET are summarized in Table 5. The key change for EET compared with ET is that $\left|V_{\text{DA}}^{\text{EET}}\right|$ for Trp7 is larger than the value for Trp14. If the intervening residues are removed from the calculation, the computed couplings are 5.68 × 10^−3^ eV and 2.56 × 10^−4^ eV for Trp7 and Trp14, respectively. This represents only a modest change in the relative coupling strengths, whereby the strength of coupling for Trp7 remains about 23 times larger than for Trp14. This suggests that the intervening residues do not play a key role in the qualitative difference in the rates of EET between the two tryptophan residues. Similar to ET, there is an increase in the strength of coupling for the *S*_1_ state compared to the *S*_0_ state for EET.

**Table 5:** Computed TDDFT (CAM‐B3LYP/6‐31G*) coupling values in eV for ET and EET for the Trp + Heme with three intervening amino acid residues model.

	Trp14 $\left(S_{0}\right)$	Trp14 $\left(S_{1}\right)$	Trp7 $\left(S_{0}\right)$	Trp7 $\left(S_{1}\right)$
$\left|V_{\text{DA}}^{\text{ET}}\right|$	$6.75\times 10^{-3}$	$6.92\times 10^{-3}$	$4.10\times 10^{-4}$	$4.87\times 10^{-4}$
$\left|V_{\text{DA}}^{\text{EET}}\right|$	$2.73\times 10^{-4}$	$3.13\times 10^{-4}$	$6.25\times 10^{-3}$	$7.08\times 10^{-3}$

**Figure 5:**
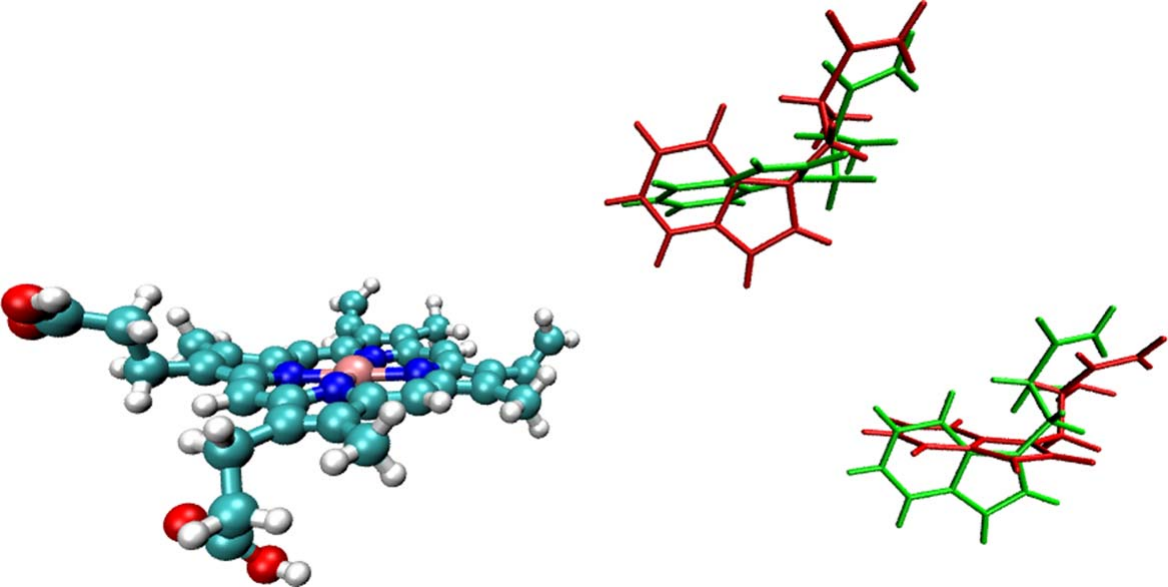
Modified heme and tryptophan system. Original tryptophan orientations shown in green and modified orientations shown in red. [Color figure can be viewed at wileyonlinelibrary.com]

Another potentially important factor that determines the strength of the coupling is the orientation of the tryptophan residues with respect to the heme. To investigate this, the strength of coupling has been computed with the Trp7 rotated such that it has the orientation of Trp14 and conversely Trp14 was rotated to have the orientation of Trp7. This is illustrated in Figure 5. This increases the coupling of “Trp14” from 2.56 × 10^−4^ eV to 2.85 × 10^−2^ eV, and decreases the coupling of “Trp7” from 5.68 × 10^−3^ eV to 7.80 × 10^−5^ eV, and thus accounts for the qualitative difference in the EET of the two residues. There is an increase in the modified coupling strengths indicating that distance from the heme does play a role, but the effect of orientation is orders of magnitude greater. This sensitivity to the orientation of the donor residues is consistent with EET occurring by a Förster mechanism.

To compute the rates ET and EET and allow a direct comparison with experiment, the reorganization energy (*λ*) and change in free energy ( ΔG) need to be determined [eq. (1)]. This requires structural optimization of the excited states corresponding to the final and initial states of the ET and EET processes. Based on optimizations of the excited states using the MOM approach with CAM‐B3LYP/6‐31G*, we computed values of ΔG and *λ* of −0.16 eV and 0.69 eV for Trp14 and −0.06 eV and 0.55 eV for Trp7 for ET, and values of ΔG and *λ* of −1.46 eV and 0.83 eV for Trp14 and −1.38 eV and 0.72 eV for Trp7 for EET. The calculated values of *λ* are of similar magnitude to those reported for other related systems.^66^

Through combining the computed ΔG and *λ* with the coupling strengths $\left|V_{\text{DA}}\right|$, the rates and relaxation times for ET and EET ( $\tau_{\text{ET}}=1/k_{\text{ET}}$ and $\tau_{\text{EET}}=1/k_{\text{EET}}$) can be evaluated, and three sets of relaxation times are given in Table 6. Overall, the calculation that most closely corresponds to experiment evaluated the relaxation time through combining the computed ΔG and *λ* with $\left|V_{\text{DA}}\right|$ computed with TDDFT for the *S*_1_ state, and this relaxation time is denoted *τ*_ES_. The remaining two relaxation times correspond to where ΔG and *λ* are combined with $\left|V_{\text{DA}}\right|$ computed with TDDFT for the *S*_0_ state of tryptophan (denoted *τ*_GS_) and with $\left|V_{\text{DA}}\right|$ evaluated using the pathways tunneling model (denoted *τ*_hybrid_). *τ*_GS_ also reproduce the experimental rates well and shows that the additional computational effort to evaluate the excited state coupling strengths could be avoided. The computed relaxation times reproduce the key observations made in the experiment. For ET, the relaxation time for Trp14 is much faster than for Trp7, and for EET the relaxation time for Trp7 is much faster than for Trp14. The qualitative description of the computed rate is heavily influenced by *V*_DA_, and it is possible to account for the experimental observations based solely on comparing coupling values, $k_{\text{ET}}\propto |V_{\text{DA}}{|}^{2}$. Addressing some of the approximations made in the calculations primarily the structural models used, neglect of entropy in ΔG and the neglect of solvent could lead to a more precise quantitative agreement with experiment.

**Table 6:** Calculated relaxation times in ps. For *τ*_ES_ the $\left|V_{\text{DA}}\right|$ is computed using TDDFT for the *S*_1_ state, for *τ*_GS_ $\left|V_{\text{DA}}\right|$ is computed using TDDFT for the *S*_0_ state and for *τ*_hybrid_ $\left|V_{\text{DA}}\right|$ is evaluated using the pathways tunneling model.

System	*τ*_ES_	*τ*_GS_	*τ*_hybrid_	Exp.
ET: Trp14	42	60	47	34
ET: Trp7	12000	32000	15000	40000
EET: Trp14	54000	70000	–	–
EET: Trp7	374	480	–	140

## Conclusions

The rates of tryptophan→heme ET and EET in myoglobin have been studied using a combination of DFT and TDDFT. These rates have been measured in recent 2D‐UV spectroscopic experiments by Chergui and coworkers^8^ providing an opportunity to assess the accuracy of different computational models and probe structural factors that affect the rates. Application of the tunneling pathways model shows that the important intermediate residues for ET are Val68 and Leu69 for Trp14 and Ile75 for Trp7, and inclusion of these residues is important in TDDFT calculations of the coupling matrix elements. Both the pathways tunneling model and TDDFT calculations correctly predict diabatic electron coupling matrix elements consistent with the rate of ET for Trp14 being greater than for Trp7. The predicted rate is greater for an initial *S*_1_ electronic state of the tryptophan donor compared to the ground state. With TDDFT it is possible to extend the study to consider EET, and the calculations correctly predict that the rate for EET is greater for Trp7.

Marcus theory calculations using the computed electron coupling elements for ET and EET combined with *λ* and ΔG evaluated from quantum chemical calculations of the appropriate excited states gives relaxation times in good agreement with experimental measurements. Subsequent analysis of the structure shows that the different rates of ET from the two tryptophan residues can be associated with the distance between the heme and tryptophan residues, while for EET the orientation of the tryptophan residues relative to the heme is important.
